# Knowledge, attitudes and practices related to schistosomiasis transmission and control in Leyte, Philippines

**DOI:** 10.1371/journal.pntd.0007358

**Published:** 2019-05-02

**Authors:** Isabel Francisco, Mario Jiz, Marieke Rosenbaum, Palmera Baltazar, Jennifer A. Steele

**Affiliations:** 1 Department of Infectious Disease and Global Health, Cummings School of Veterinary Medicine at Tufts University, Grafton, Massachusetts, United States of America; 2 Department of Immunology, Research Institute for Tropical Medicine, Muntinlupa, Metro Manila, Philippines; University of Queensland School of Veterinary Science, AUSTRALIA

## Abstract

Schistosomiasis is a chronic but preventable disease that affects 260 million people worldwide. In the Philippines, 860,000 people are afflicted with *Schistosoma japonicum* annually, and another 6.7 million live in endemic areas. The disease’s complex epidemiology as well as the influence of poverty in endemic areas demand an integrated, multi-sectoral approach to disease control. Results from behavioral or sociocultural studies on schistosomiasis could improve the content and impact of schistosomiasis control in rural villages in the Philippines. We investigated knowledge, attitudes and practices related to schistosomiasis transmission and control in an endemic village in Leyte Province, Philippines. We administered a questionnaire to 219 participants covering 1) knowledge and attitudes related to schistosomiasis, its symptoms, and its transmission; 2) attitudes and practices in relation to schistosomiasis prevention; 3) willingness to comply with public health control programs; and 4) whether the respondent had previously contracted schistosomiasis. Responses revealed fairly high measures of schistosomiasis knowledge (mean 17.0 out of 23 questions, range 6–23), but also inconsistent disease prevention behavior. A high proportion of participants (72.6%, n = 159) reported previous disease. Participant belief in the preventability of schistosomiasis was revealed to be a key attitude, as carabao owners who believed in prevention were over five times more likely to be willing to vaccinate their carabaos (OR = 5.24, 95% CI 1.20–27.68, *P* = 0.04). Additionally, participants who did not believe in prevention were about twice as likely to report previous disease (OR = 2.31, 95% CI 1.02–5.63, *P* = 0.05). Our results suggest that future public health interventions should address barriers to disease-preventing behavior, as well as maintaining community belief in disease prevention. Comprehensive disease control programs should be supplemented by sociocultural and behavioral context in order to improve their impact in endemic communities.

## Author summary

Schistosomiasis is a chronic tropical disease caused by parasitic worms of the genus *Schistosoma*. In the Philippines, *Schistosoma japonicum* afflicts over 800,000 people annually, and another 6.7 million live in endemic areas. The current national control program based on human mass treatment has been unable to eliminate schistosomiasis on its own. This is in part due to the poverty of endemic areas as well as the parasite’s complex life cycle, which includes transmission by freshwater snails and livestock. A more comprehensive disease control strategy would include periodic treatment and vaccination of the widely used domestic water buffalo or carabao. Sociocultural and behavioral research would also inform and improve the impact of future control programs. To this end, we investigated knowledge, attitudes, and practices related to schistosomiasis in an endemic village in Leyte, Philippines. We found high levels of disease knowledge and awareness, but also inconsistencies in disease prevention habits. Belief in whether schistosomiasis could be prevented was important in this community, associated with previous disease experience as well as receptivity to new strategies like livestock vaccination. This kind of social context is vital to future public health campaigns, and highlights the importance of cross-disciplinary work to achieve successful disease control.

## Introduction

Schistosomiasis is a chronic disease caused by trematode worms of the genus *Schistosoma* and transmitted by freshwater snails [1]. In 2013, the World Health Organization (WHO) estimated 260 million people at risk for the disease [2]. The total burden of disease attributed to the three major species, *Schistosoma japonicum*, *Schistosoma mansoni*, and *Schistosoma haematobium*, has been estimated at 24 to 29 million disability adjusted life years [3]. *S*. *japonicum*’s zoonotic nature is key to its transmission, as the snail intermediate host *Oncomelania hupensis quadrasi* is required to release cercaria for transcutaneous infection [4]. Once present in freshwater, cercariae penetrate skin and migrate to the portal vessels, where they mature, pair with the opposite sex, and produce eggs to be shed in feces [4]. In this way multiple mammalian species may propagate transmission of the disease as long as water contact occurs.

In the Philippines, schistosomiasis, caused by *S*. *japonicum*, afflicts 860,000 people annually [2]. Another 6.7 million people live in endemic areas, with major endemic foci in the poorest regions of the Visayas and Mindanao [5]. National control strategies were initially directed at snail population control due to the lack of an effective drug [4, 6]. When the oral chemotherapeutic praziquantel (PZQ) was introduced in 1979, the focus shifted to morbidity reduction using case-by-case and mass treatment with PZQ [4, 6]. Although disease prevalence dropped dramatically in the 1980s, human mass treatment was unable to eliminate schistosomiasis on its own [6, 7]. Additionally, mass treatment programs were largely sustained by temporary external funding, particularly a loan from the World Bank that ceased in 1995 [8].

Control of human infection is complicated by the parasite’s persistence in animal reservoirs such as the domestic water buffalo or carabao, with prevalence in carabao reported as high as 52% [6, 9]. Additionally, poverty in endemic communities fosters disease prevalence by limiting access to health care and increasing exposure to contaminated water [3]. Periodic treatment and vaccination of carabaos are now being evaluated by the Research Institute for Tropical Medicine (RITM) as control strategies due to the domestic species’ role as a major reservoir host [6, 8–12]. Combined with mass treatment and snail control, this approach could lead to more sustainable and efficient disease control in the Philippines.

However, studies at the RITM have identified a lack of behavioral and sociocultural research to supplement these interventions [13]. Concern for the disease is low in rural communities, where locals shun the use of boots in traversing potential schistosome habitats [1]. When faced with losing their livelihoods, infected rice farmers will often continue to work rather than seek treatment or decrease their water contact [1]. Additionally, mass treatment programs face problems of compliance, with endemic communities preferring case-by-case treatment over mass treatment [1]. Refusal to take the drug when symptoms are absent is rooted in misconceptions about the disease and fear of side effects or toxicity [1, 13]. A more complex, multi-sectoral approach to schistosomiasis control is necessary in order to properly address both the biology and epidemiology of the disease [3, 5, 14].

Examining a community’s knowledge, attitudes, and practices (KAP) around schistosomiasis could highlight previously unrecognized limitations to current control efforts, including knowledge gaps, cultural beliefs, or behaviors. Results from behavioral or sociocultural research could therefore inform the specific content and format of future public health interventions to improve the impact of disease control in endemic areas. To our knowledge, this is the first KAP study on schistosomiasis in this area despite years of government interventions, biomedical research, and disease endemicity. To this end, we assessed knowledge, attitudes and practices surrounding schistosomiasis transmission and control in an endemic village in Leyte, Philippines. We also investigated the effect of schistosomiasis knowledge, such as knowledge of symptoms and transmission, on behaviors and attitudes related to disease control. These included individual disease prevention behaviors as well as willingness to comply with mass treatment or carabao vaccination. Finally, we identified respondent characteristics that were predictive of these attitudes, as well as characteristics that were predictive of having contracted schistosomiasis in the past.

## Methods

### Study site and participants

This study was conducted in Macanip, a local government unit or “barangay” in the municipality of Jaro, Leyte. Leyte is an Eastern Visayas province of the Philippines, and is one of the major endemic foci of schistosomiasis [5]. According to the 2010 census by the Philippine National Statistics Office, the population of Macanip was 1,755 [15]. The RITM has conducted other schistosomiasis research in the area, including bovine vaccine trials. Macanip is loosely organized into three zones located in the town center, and eight sitios or territories distanced from it. All three zones and all eight sitios were included in the study, and each zone or sitio was sampled proportional to its number of households.

The minimum sample size calculated based on the primary study objective to assess knowledge, attitudes, and practices in the community was 96 participants (95% CI = 95.98–96.02, ME = 10%), based on the 2010 population size, a prevalence of 50%, and 80% power. No data for schistosomiasis prevalence in Macanip within the last 10 years were published at the time of the study. In each zone or sitio, participants were recruited by randomly selecting households on a predetermined walking route, and then knocking on doors. Questionnaires were administered via oral interview to one adult (18 years of age or older) in each selected household. In each household only the first participant to consent to the study was interviewed.

This study was approved by the Institutional Review Board (IRB) of the Tufts Medical Center / Tufts University Health Sciences in Boston, MA (IRB#11699) and the Research Institute of Tropical Medicine (RITM) in Manila, Philippines (IRB #2015–11). The study qualified for exemption status with the Tufts IRB. All relevant research procedures adhered to the guidelines of both institutions, including the use of oral consent for data collection. Village officers were asked for permission prior to initiation of the study. All participants were orally informed of the purpose and procedures of the study, with emphasis on voluntary participation and confidentiality. All participants were also provided with an information sheet satisfying the basic elements of informed consent, translated into Waray-waray and verbally communicated by community health workers. Only participants who verbally consented to the study after considering this sheet were included.

### Study design

This study was analytic and cross-sectional in design and employed the use of a standardized questionnaire. The questionnaire was designed based on consultation with researchers from both the Cummings School of Veterinary Medicine at Tufts University and the RITM. The questionnaire included both open- and closed-ended questions and was designed to take no more than 30 minutes to administer. The questionnaire was written in English, translated to Waray-waray by RITM staff who had experience working in the area, and back-translated to English for IRB approval. Questions covered 1) knowledge and attitudes related to schistosomiasis, its symptoms, and its transmission; 2) attitudes and practices in relation to schistosomiasis prevention; 3) willingness to comply with public health control programs such as mass treatment and carabao vaccination; and 4) whether the respondent had contracted schistosomiasis in the past. Additional questions covered socioeconomic characteristics, medical history and relevant household conditions such as access to different local water sources and sanitary facilities.

Questionnaires were administered verbally by six RITM staff members who had previous experience assisting with survey-based schistosomiasis research in Macanip. Due to other RITM studies, all interviewers had previously received training on maintaining confidentiality and administering questionnaires in a standardized manner. During the study, each interviewer was accompanied by one Barangay Health Worker (BHW), a community health worker trained by the Philippine Department of Health. BHWs did not participate in the interviews, but assisted in navigating the area, locating households and greeting participants. All six interviewers spoke Waray-waray and Tagalog fluently, as well as basic English. Interviews primarily followed the written protocol in Waray-waray, with some flexibility allowed in clarifying participants’ answers with additional questions. For open-ended questions, answers were written down word-for-word in Waray-waray and then translated to English by RITM staff on the same day. Participants were not paid monetarily but were informed that the results of the study would be made available to them via local control programs as well as RITM-led meetings.

### Data processing and statistical analysis

‘Knowledge scores’ were calculated from questions on knowledge of schistosomiasis symptoms, transmission and prevention using the sum of total correct responses. ‘Prevention scores’ were similarly calculated from questions related to engagement in prevention strategies that are recognized to be effective (scored as correct or 1) and in strategies that are recognized as ineffective (scored as incorrect or 0). For example, a participant who answers yes to whether she avoids water contact and also answers yes to whether she visits a traditional healer is awarded one point to her prevention score, for avoiding water contact. Answers to open-ended questions were qualitatively grouped into common themes and counted by theme. These included questions such as where the participant had first heard about schistosomiasis.

Data analysis was conducted using R v 3.3.1. The data were found to be non-normally distributed, and the following analyses were conducted to determine significant associations between outcomes of interest and predictor variables: 1) Spearman’s correlation was performed to determine the relationship between knowledge score and prevention score; 2) Mann-Whitney U test was used to assess significant differences in knowledge scores between those willing or unwilling to participate in mass treatment in the future; 3) Mann-Whitney U test was also used to assess differences in knowledge scores between carabao owners willing or unwilling to vaccinate their carabaos; and 4) multiple logistic regressions were used to identify factors predictive of 3 different outcome variables. These outcome variables included a) willingness to participate in mass treatment in the future; b) willingness to vaccinate carabaos, with only carabao owners included; and c) having contracted schistosomiasis in the past. A *p*-value of ≤0.05 was accepted as statistically significant for all analyses.

### Multiple logistic regression

Predictive models for the three outcome variables described above (a, b, and c) were developed using multiple logistic regression. For each regression, predictor variables were selected by performing a univariate analysis of each variable with the outcome of interest. These variables included demographic data, knowledge score, prevention score, household conditions such as presence of a working toilet, and attitudes around schistosomiasis such as belief that the disease can be prevented. Variables also included whether participants had heard of schistosomiasis, whether they had learned about it in school, and whether they described schistosomiasis as common to the area. A p-value of ≤0.10 was used to initially determine inclusion in the first model. Next, a second model was developed, including all predictor variables that had a p-value of ≤0.05 in the first model. Each of the remaining variables that had been included in the first model but not the second (i.e. those with a p-value between 0.05–0.10) were then added back individually and tested for significance. The final model included all variables that remained significant in this second model, as well as the demographic variables of age, sex, and education level. A p-value of ≤0.05 was used for statistical significance for the final model.

## Results

In total, 219 interviews from distinct households were conducted in July of 2015. The study included 97 male and 122 female participants, ranging in age from 19 to 84 years old (mean = 48). All participants spoke Waray-waray, the local language in the Eastern Visayas region, and many participants were bilingual or trilingual. All interviews except one were conducted in Waray-waray; one participant was trilingual and preferred the Tagalog dialect, which is the Philippine national language.

Table 1 describes demographic characteristics as well as any self-reported history of being diagnosed with schistosomiasis, or activities recognized as risk factors for the disease. The mean household size was 4.5 (range 1–14), and the majority of households (86.8%, n = 190) had a total monthly income under PHP2,000.00 (42.00USD). The majority of participants (42.9%, n = 94) were employed as farmers, 72.6% (n = 159) had previously contracted schistosomiasis, and 86.8% (n = 190) had previously participated in the mass treatment program. More than half (62.2%, n = 102) of participants who had contracted schistosomiasis and pursued treatment for the disease said that they were treated at no cost through a mass treatment program. The majority of participants (85.8%, n = 188) said they had regular water contact, most commonly through chores such as washing clothes (64.8%, n = 142) or through their occupation e.g. rice farming (54.3%, n = 119).

**Table 1:** Socio-demographic characteristics of study participants (N = 219) in Barangay Macanip, Leyte, Philippines, where schistosomiasis is endemic.

Characteristic	No. (%)
Age of respondent	
18–24	10 (4.57)
25–44	88 (40.18)
45–64	82 (37.44)
65+	39 (17.80)
Gender	
Male	97 (44.29)
Female	122 (55.71)
Educational status	
Tertiary	17 (7.76)
Secondary	86 (39.27)
Primary	113 (51.60)
No formal education	2 (0.91)
Did not specify	1 (0.46)
Animals owned	
Chicken	154 (70.32)
Pig	125 (57.08)
Dog	98 (44.75)
Cat	75 (34.25)
Carabao (water buffalo)	48 (21.92)
Goat	2 (0.91)
Other	6 (2.74)
Previously contracted schistosomiasis	159 (72.60)
If treated, did so through mass treatment	102 (62.20)
Previously participated in mass treatment	190 (86.76)
Reported regular contact with local bodies of water	188 (85.84)
Have access to a working toilet	159 (72.60)

Table 2 describes the knowledge, attitudes and practices of participants in relation to schistosomiasis transmission, infection, and prevention. The mean knowledge score was 17.0 out of 23 questions (range 6–23). All participants interviewed had heard of schistosomiasis before, and almost all (99.1%, n = 217) described it as common to their village. The majority of participants had first learned about schistosomiasis from existing control programs in the village (51.1%, n = 112) or from previous research conducted by the RITM (37.0%, n = 81). Almost half (47.5%, n = 104) did not remember schistosomiasis being mentioned in school at all. Most participants (90.0%, n = 197) were able to identify snails as an intermediate host or source of the disease, but only 24.2% (n = 53) were aware of a microscopic agent involved. All participants were able to identify at least one main clinical sign of schistosomiasis in humans, with paralysis and convulsions being the most commonly identified symptom (95.0%, n = 208). Over three quarters of participants (76.7%, n = 168) were aware of schistosomiasis transmission from animals to humans.

**Table 2:** Participant knowledge, attitudes and practices related to schistosomiasis in Barangay Macanip, Leyte, Philippines, where schistosomiasis is endemic.

**Knowledge parameter**^a^	**No. (%)**
First source of schistosomiasis knowledge	
Public health programs	112 (51.14)
Previous RITM research	81 (36.99)
Doctor or hospital	20 (9.13)
School	4 (1.83)
Word of mouth	2 (0.91)
Aware of zoonotic transmission of schistosomiasis	168 (76.71)
Awareness of infection sources of schistosomiasis	
Microscopic agent	53 (24.20)
Snails	197 (89.95)
Awareness of schistosomiasis transmission	
Working in rice fields	213 (97.26)
Urinating and defecating near water source	172 (78.54)
Walking barefoot	209 (95.43)
Swimming or playing in bodies of water	207 (94.52)
Washing or bathing in bodies of water	211 (96.35)
Awareness of schistosomiasis prevention	
Avoid contact with bodies of water	197 (89.95)
Participate in mass treatment regularly	205 (93.61)
Use clean water for drinking and washing	199 (90.87)
Wear protective gear in infected water	194 (88.58)
Number of correct clinical signs identified, median (IQR)	6 (2.00)
**Attitude parameter**^b^	**No. (%)**
Belief that schistosomiasis can be prevented	
Yes	131 (59.82)
No	88 (40.18)
Perceived seriousness of schistosomiasis	
Very serious	147 (67.12)
Of some concern	57 (26.03)
Not very serious	9 (4.11)
Not serious at all	5 (2.28)
Did not answer	1 (0.46)
Willingness to participate in mass treatment	
Yes	187 (86.18)
No/not sure	30 (13.70)
Did not answer	2 (0.91)
Willingness to vaccinate carabao, carabao owners only^c^	
Yes	24 (50.00)
No/not sure	18 (37.50)
Did not answer	6 (12.50)
**Practice parameter**^d^	**No. (%)**
Correct schistosomiasis prevention practices	
Avoids contact with bodies of water	128 (58.45)
Participates in mass treatment regularly	183 (83.56)
Uses clean water for drinking and washing	193 (88.13)
Wears protective gear in infected water	147 (67.12)

^a^N = 219

^b^N = 215

^c^Only participants with one or more carabaos were included (N = 48)

^d^N = 219

Assessment of attitudes toward schistosomiasis, including willingness to comply with mass treatment and carabao vaccination programs, showed that the majority of participants (67.1%, n = 147) described schistosomiasis as a very serious disease, but 40.2% (n = 88) of participants did not believe that schistosomiasis was actually possible to prevent. Most participants (86.2%, n = 187) were willing to participate in mass treatment, but only 50% (n = 48) of carabao owners were willing to vaccinate their carabaos. Common self-reported reasons for not participating in mass treatment were old age (36.7%, n = 11) and fear of side effects, allergic reactions or toxicity (20.0%, n = 6). The most common reason for carabao owners refusing carabao vaccination was fear of side effects or fear for the carabao’s health due to its importance in the family’s livelihood (55.5%, n = 10).

The average prevention score based on assessment of practices was 2.97 out of 4 correct practices listed (range 0–4). The most common correct practice reported was using clean water for drinking and washing (88.1%, n = 193). Despite very high awareness of prevention practices as shown in Table 1, only 58.5% (n = 128) said they actively avoided contact with bodies of water, and only 67.1% (n = 147) said they used protective gear such as boots in infected water. Almost half of participants (45.2%, n = 99) said they used herbal treatments to maintain wellness, and 35.6% (n = 78) said they visited the traditional healer to maintain wellness. Spearman’s correlation demonstrated no significant association between knowledge and prevention scores. Knowledge scores were not significantly different between those willing or unwilling to comply with mass treatment or carabao vaccination programs.

Table 3 shows factors predictive of willingness to participate in mass treatment programs in the future. Age had an inverse association with willingness to participate in mass treatment programs (odds ratio [OR] = 0.97, 95% CI 0.94–0.99, *P* = 0.02). Additionally, participants who had contracted schistosomiasis in the past were nearly 6 times more likely to be willing to participate in mass treatment programs (OR = 5.91, 95% CI 2.57–14.15, *P* < 0.001). Table 4 shows factors predictive of willingness to vaccinate carabaos for schistosomiasis, with only carabao owners (n = 42) included in this regression. Carabao owners who believed schistosomiasis could be prevented were over five times more likely to be willing to vaccinate their carabao(s) (OR = 5.24, 95% CI 1.20–27.68, *P* = 0.04). Prevention scores did not significantly vary with this belief in prevention of the disease. Additionally, carabao owners who were willing to participate in mass treatment programs were over 20 times more likely to be willing to vaccinate their carabao(s) (OR = 22.12, 95% CI 2.12–649.14, *P* = 0.03).

**Table 3:** Factors predicting respondent willingness to participate in future mass treatment programs in Barangay Macanip, Leyte, Philippines, where schistosomiasis is endemic (multiple logistic regression).

Variable	Odds ratio*	95% CI	p-value
Age (continuous)	0.97	0.94–0.99	0.02
Has previously contracted schistosomiasis			
Yes	5.91	2.57–14.15	<0.001
No	1.00		

*adjusted for respondent sex and education level

**Table 4:** Factors predicting carabao owners’ willingness to vaccinate their carabaos for schistosomiasis in Barangay Macanip, Leyte, Philippines, where schistosomiasis is endemic (multiple logistic regression)^a^.

Variable	Odds ratio*	95% CI	p-value
Believes that schistosomiasis can be prevented			
Yes	5.24	1.20–27.68	0.04
No	1.00		
Willing to participate in mass treatment in the future			
Yes	22.12	2.12–649.14	0.03
No	1.00		

*adjusted for respondent age, sex, and education level

^a^only including those who owned one or more carabaos and who reported willingness to vaccinate (N = 42)

Finally, Table 5 shows the four variables that remained in the final model for having previously contracted schistosomiasis. Participants who did *not* believe that schistosomiasis could be prevented were about twice as likely to have contracted the disease in the past (OR = 2.31, 95% CI 1.02–5.63, *P* = 0.05). Participants who had access to a working toilet at home were over twice as likely to have previously contracted the disease (OR = 2.55, 95% CI 1.08–6.00, *P* = 0.03). If participants had previously participated in mass treatment programs at least once before, they were over 100 times more likely to have contracted schistosomiasis at least once before (OR = 105.89, 95% CI 18.6–2041.58, *P* < 0.001). Additionally, those that reported that they were willing to participate in mass treatment programs in the future were nearly 5 times more likely to have previously contracted the disease (OR = 4.53, 95% CI 1.53–13.41, *P* = 0.006). For all 3 logistic regressions, factors remained significantly predictive when the model was adjusted for the demographic variables of age, sex, and education level.

**Table 5:** Factors predicting which of 219 respondents had previously contracted schistosomiasis in Barangay Macanip, Leyte, Philippines, where schistosomiasis is endemic (multiple logistic regression).

Variable	Odds ratio*	95% CI	p-value
Believes that schistosomiasis can be prevented			
Yes	1.00		
No	2.31	1.02–5.63	0.05
Has access to working toilet at home			
Yes	2.55	1.08–6.00	0.03
No	1.00		
Has previously participated in mass treatment			
Yes	105.89	18.6–2041.58	<0.001
No	1.00		
Willing to participate in mass treatment in the future			
Yes	4.53	1.53–13.41	0.0058
No	1.00		

*adjusted for respondent age, sex, and education level

## Discussion

### Knowledge

All participants had heard of schistosomiasis, and almost all participants knew it was common to the area. These findings suggest high awareness of the disease in Macanip. As well as being endemic to Leyte, schistosomiasis has necessitated multiple research projects by RITM staff, which may account for such high awareness. Additionally, 72.6% of participants had previously contracted schistosomiasis, further confirming endemicity of the disease. Knowledge scores were fairly high, and participants demonstrated accurate knowledge of transmission, clinical signs and proper prevention techniques (Table 2). However, most participants were unaware of a microscopic agent causing the disease (Table 2). Additionally, almost half the participants did not remember learning about schistosomiasis in school, with most citing existing RITM programs or previous research as the source of their knowledge (Table 2). The high prevalence of disease and long history of control efforts in Macanip could have necessitated basic knowledge of schistosomiasis. However, our findings suggest limited understanding of the biology of the disease. Improving the quality of disseminated information might encourage compliance with disease control efforts, as well as preventing misconceptions about mass treatment or other interventions.

### Attitudes

Our study also reveals important attitudes surrounding schistosomiasis control and the effects of these attitudes on receptiveness to control programs. Most participants considered schistosomiasis to be a very serious disease, and expressed willingness to participate in mass treatment, suggesting high compliance in future mass treatment programs (Table 2). However, research in Samar, the endemic province closest to Leyte, has showed far lower compliance on the day of mass treatment than predicted from surveys [13]. Further study is therefore needed on how willingness to participate can be translated into actual compliance in mass treatment.

Additionally, only half the carabao owners in this study expressed willingness to have their carabaos vaccinated for schistosomiasis (Table 2). The most frequently cited reason for being unwilling was fear for the carabao’s health, suggesting misconceptions and doubts surrounding the idea of livestock vaccination. Knowledge scores did not vary significantly between participants who were willing and unwilling to participate in mass treatment or between owners willing and unwilling to vaccinate their carabaos. There is therefore no evidence that current knowledge of the disease affects willingness to comply with these control programs. Knowledge alone may therefore be an insufficient motivator for compliance in this community.

Instead, belief in preventability of the disease was revealed to be a key attitude in this study. About 60% of participants believed it was possible to prevent schistosomiasis, while 40% of participants did not (Table 2). These findings suggest that a significant proportion of Macanip residents do not view schistosomiasis as a solvable problem for their community. This most likely stems from the persistence of schistosomiasis in Macanip despite decades of control measures. However, carabao owners that did believe in prevention were over five times more likely to be willing to vaccinate their carabaos (Table 4). This finding suggests that those who believe in disease prevention are more open to new strategies for disease control. The success of future interventions in Macanip may therefore rely on preserving this attitude within the community via health promotion. Efforts to educate residents on the importance of water contact, for example, should emphasize that this practice makes it possible to be free of schistosomiasis.

We also found that age had a significant effect on attitudes about disease control, where older participants were less likely to be willing to participate in future mass treatment programs (Table 3). Additionally, carabao owners who were willing to participate in future mass treatment were much more willing to have their carabaos vaccinated (Table 4). These findings once again highlight the influence of sociocultural context on the impact of public health interventions. If compliance with disease control tends to decrease with age, targeting an older cohort may improve the impact of future control efforts. Mass treatment campaigns, for example, should be expanded toward older members of the community in messaging and accessibility. Additionally, if receptiveness to different aspects of disease control are interrelated, and if belief in prevention plays a role, then interventions must adequately address these attitudes in order to succeed. To this end, regular recruiting efforts for mass treatment could also disseminate media aiming to positively influence participant attitudes, such as belief in prevention.

### Practices

Prevention scores were high among participants, but our findings indicate inconsistent behavior around disease prevention. Belief in the preventability of schistosomiasis was not found to significantly impact prevention score. However, around 40% of participants said they did not actively avoid water contact, and more than 30% said they did not use protective gear while contacting water, despite high awareness of both these prevention strategies (Table 2). Additionally, a large majority of participants (85.8%) reported regular water contact, despite about 60% saying they actively tried to avoid it (Tables 1 and 2). This discrepancy suggests that Macanip residents are aware of disease prevention or might believe in its efficacy, but still encounter barriers to it in practice. During interviews, participants mentioned neglecting these strategies when they were not practical, such as declining to wear heavy boots while working in a rice field for many hours. Some participants mentioned using herbal treatments and visiting the traditional healer as health-seeking behaviors, but spoke in the context of maintaining wellness rather than treating schistosomiasis. These anecdotes suggest further obstacles to disease prevention that Macanip residents may encounter, and reinforce the need for behavioral and sociocultural research related to this disease. Identifying the community’s unique barriers to disease prevention via qualitative research would allow for more effective design of public health interventions or recommendations.

### Previous disease

Our study found a high proportion of participants (72.6%) who reported that they had previously contracted schistosomiasis (Table 1). Neither knowledge score nor prevention score was found to have a significant effect on previous disease, implying that current levels of awareness and disease-preventing behavior may not be related to previous experience with schistosomiasis. Additionally, respondents who had access to a working toilet at home were more likely to report previous disease (Table 5). Although this finding is unexpected, participants were not asked when they had acquired their toilet in relation to contracting schistosomiasis. Appropriate waste disposal may therefore be insufficiently protective of disease in this setting, and those who experience the disease may then become motivated to acquire a toilet in the home.

Once again, belief in the preventability of schistosomiasis was a key attitude, with participants who did not believe in prevention being more likely to report previous disease (Table 5). Although this attitude did not have a significant effect on prevention score, belief in prevention might impact behavior in other ways, such as decreasing the consistency or effort behind prevention practices. Conversely, participants might cease to believe that schistosomiasis is preventable after experiencing the disease themselves, in a community where it is highly prevalent. Further research is needed to investigate the relationship of this attitude to disease status and prevention behavior.

Finally, attitudes and practices around mass treatment programs were also associated with contracting schistosomiasis. Those who reported willingness to participate in future mass treatment were more likely to report previous disease. Additionally, those who reported previous participation in mass treatment were much more likely to report previous disease (Table 5). This association is consistent with our finding that over half the participants who sought schistosomiasis treatment in the past did so through mass treatment programs (Table 1). Those who contract schistosomiasis might therefore pursue mass treatment, and then continue to view it favorably once treated successfully. Ultimately, mass treatment appears to be a well-established and popular form of health care in Macanip, but fails to fully control schistosomiasis. This status quo of well-known but insufficient care might have shaped community attitudes on current disease control as well as receptiveness to new interventions.

### Limitations

This study was most significantly limited by the format of the KAP questionnaire. Although attempts were made to ask open-ended questions, most of our data relied upon dichotomous and numerical observations. This was especially relevant to prevention practices, where many participants with high prevention scores still reported frequent water contact. Further studies should aim for more open-ended assessment of disease prevention, including more qualitative data on barriers to behavior change, and incorporate an observational component to validate questionnaires. Additionally, our sampling strategy may have created bias by selecting households upon a predetermined walking route during the work day. Macanip residents who lived farther from main pathways or worked away from home during the day may have been excluded by convenience sampling. These limitations in study design stem from conducting the first attempt at KAP research in this area on a limited timeline and budget. We invite further studies to spend more time recruiting participants and using more open-ended methods to assess knowledge, attitudes, and practices.

Our study aimed to quantify self-reported disease alongside attitudes on disease prevention. However, future research in Macanip should continue to monitor adult human prevalence when possible, using screening tests such as Kato-Katz stool examination. Because schistosomiasis has been prevalent for so many years, future studies should also obtain temporal data on disease prevalence, as much of our data may have been shaped by previous medical histories.

### Conclusion

Our study revealed fairly high levels of awareness and good baseline knowledge of schistosomiasis in Macanip, as well as frequent attempts at disease-preventing behavior. These findings serve as a testament to the many years of research, training, and disease control programs in Macanip. However, we found inconsistencies in prevention practices that suggest barriers to lasting behavior change. Future interventions in Macanip should address specific obstacles to disease-preventing behavior via further qualitative research.

Our results highlight belief in the preventability of schistosomiasis as a pivotal attitude, influencing participant willingness to vaccinate livestock as well as their likelihood of reporting previous disease. Our findings imply that belief in prevention may be associated with more receptiveness to new control strategies such as carabao vaccination. Future public health interventions could therefore include efforts to bolster this belief, such as providing examples of other communities that have successfully lowered their burden of disease.

Recent studies from the People’s Republic of China are often referenced as success stories to this end. In 2004, China implemented an integrated strategy for schistosomiasis control that includes mass treatment and education but also snail control, improved sanitation, and replacement of working bovines with agricultural machinery [16, 17]. This shift has resulted in the elimination of human schistosomiasis cases in formerly endemic communities, as well as the elimination of infected bovines and snails in selected areas [16, 17]. However, it is important to note that most rural endemic communities such as Macanip suffer from long-term poverty as well as disease. Local poverty fosters disease transmission by increasing exposure to contaminated water as well as limiting the means to treat or prevent disease [3]. Interventions that target the parasite’s life cycle (e.g. bovine vaccination) should therefore be employed alongside strategies for poverty reduction [18]. Ultimately, effective disease control requires a long-term multi-sectoral commitment toward breaking the cycle of *S*. *japonicum* transmission as well as mitigating the effects of poverty on public health outcomes.

Our findings emphasize the importance of cross-disciplinary work to successful schistosomiasis control in Macanip and in similar endemic communities. Studies that provide sociocultural and behavioral data should continue to supplement biomedical research in shaping future interventions. This focus on a comprehensive, integrated approach is relevant to similar endemic communities currently designing and implementing programs for the control and elimination of schistosomiasis.

## Supporting information

S1 Checklist STROBE Checklist. (DOC)

S1 Table Raw data table. (XLSX)
